# Construction of uricase-overproducing strains of *Hansenula polymorpha *and its application as biological recognition element in microbial urate biosensor

**DOI:** 10.1186/1472-6750-11-58

**Published:** 2011-05-25

**Authors:** Kostyantyn V Dmytruk, Oleh V Smutok, Olena V Dmytruk, Wolfgang Schuhmann, Andriy A Sibirny

**Affiliations:** 1 Institute of Cell Biology, NAS of Ukraine, Drahomanov Street 14/16, Lviv 79005, Ukraine; 2 Analytische Chemie - Elektroanalytik & Sensorik, Ruhr-Universitat Bochum, Universitätsstr. 150, D-44780 Bochum, Germany; 3 Department of Biotechnology and Microbiology, Rzeszow University, Cwiklinskiej 2, 35-601 Rzeszow, Poland

**Keywords:** urate, oxidoreductase, methylotrophic yeast, amperometric biosensor, cell-based biosensor

## Abstract

**Background:**

The detection and quantification of uric acid in human physiological fluids is of great importance in the diagnosis and therapy of patients suffering from a range of disorders associated with altered purine metabolism, most notably gout and hyperuricaemia. The fabrication of cheap and reliable urate-selective amperometric biosensors is a challenging task.

**Results:**

A urate-selective microbial biosensor was developed using cells of the recombinant thermotolerant methylotrophic yeast *Hansenula polymorpha *as biorecognition element. The construction of uricase (UOX) producing yeast by over-expression of the uricase gene of *H. polymorpha *is described. Following a preliminary screening of the transformants with increased UOX activity in permeabilized yeast cells the optimal cultivation conditions for maximal UOX yield namely a 40-fold increase in UOX activity were determined.

The UOX producing cells were coupled to horseradish peroxidase and immobilized on graphite electrodes by physical entrapment behind a dialysis membrane. A high urate selectivity with a detection limit of about 8 μM was found.

**Conclusion:**

A strain of *H. polymorpha *overproducing UOX was constructed. A cheap urate selective microbial biosensor was developed.

## Background

Urate is the final purine intermediate of the purine nucleotide catabolism in human [1]. Normally, urate is not accumulated in human body fluids and hence its concentration is a valuable indicator in clinical diagnosis [2] indicating gout, hyperuricemia, or Lesch-Nyhan syndrome [3]. Elevated urate levels are also related to e.g. increased alcohol and cholesterol consumption, obesity, diabetes, kidney and heart diseases, and they are considered to be a risk factor for cardiovascular diseases [4].

Uricase (urate oxidase, UOX, EC 1.7.3.3) is a key enzyme in the purine degradation pathway. It catalyzes the oxidation of urate in the presence of oxygen, producing allantoin and CO_2_. Uricase does not require an additional cofactor other than oxygen for the enzymatic reaction and is therefore widely used for the amperometric determination of urate to diagnose gout and the above mentioned diseases and conditions. For urate determination in serum and urine, several biosensors have been reported [5-8]. Numerous amperometric urate biosensors based on uricase or uricase coupled with peroxidase varying in the type of electrode, immobilization technique, ability and type of redox mediator(s), conductive polymers *etc. *have been described [9-15].

Several new amperometric uric acid biosensors were fabricated by immobilizing uricase onto gold nanoparticles or carbon nanotubes [16-18]. These urate-selective biosensors are using expensive commercially available uricases purified from *Bacillus fastidiosus, Arthrobacter globiformis*, or *Candida utilis*. Therefore, the fabrication of cheap urate-selective amperometric biosensors is still a challenging task.

In this work, we introduce a novel microbial urate-selective amperometric biosensor based on the cells of the uricase-overproducing recombinant yeast *H. polymorpha*.

## Results and Discussion

### Construction of the uricase-overproducing recombinant yeast H. polymorpha

Uricase-overproducing transformants of *H. polymorpha *were constructed using straight-forward microbiological techniques. The expression of target genes and corresponding enzymatic activity in yeasts is increased by repeated amplification of the required nucleotide sequence [19,20]. The plasmid pGLG61 contains a bacterial *APH *gene (aminoglucoside-3-phosphotransferase) as dominant marker whose expression is impaired, and the sequence of the *HARS36 *(*TEL188*) autonomic replicating sequence. This ensures multiple tandem integration of pGLG61 into the *H. polymorpha *telomere regions on medium containing the antibiotic G418 [21].

**Figure 1:**
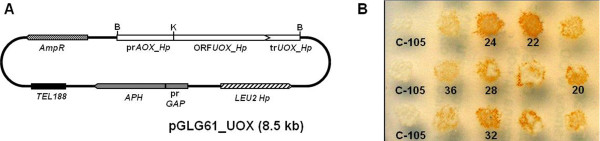
**A: Circular scheme of the plasmid pGLG61_UOX (8.5 kb)**. The *HpAOX *promoter and *UOX *ORF with terminator region are shown as open boxes. The *LEU2 *gene *H. polymorpha *is shown as hatched box. Gene *AmpR *conferring resistance to ampicillin is shown as chequered boxes. The *H. polymorpha *truncated glyceraldehyde-3-phosphate dehydrogenase (*GAP*) promoter and the geneticin resistance gene (*APH*) are shown as grey boxes, and the tellomeric region (*TEL188*) as black box. Restriction sites: B, *Bam*HI; K, *Kpn*I. **B**: Visualization of UOX activity *in situ *in cells grown on mineral medium plates supplemented with glucose, incubated for 18 h, and then overlain with UOX reaction mixture with permeabilizing agent as described in Materials and Methods section.

The recombinant plasmid pGLG61_UOX (Figure 1A) derived from pGLG61 was transformed to the recipient strain *H. polymorpha *C-105 (*gcr1*, *cat*Х), impaired in glucose repression and devoid of catalase activity. The transformants were grown on YPS medium in the presence of increasing concentrations of G418. The highest concentration of G418 which allowed the transformants to grow was 1 mg mL^-1^. The transformants were stabilized by cultivation in non-selective media for 10-12 generations with further shifting to the selective media with G418. The presence of the expression cassette in the stable transformants was examined by diagnostic PCR using the primers Ko183/Ko190 and the genomic DNA of stable transformants as a template. Fragments of predictable size (~2.7 kb) were obtained (data not shown).

The parental strain С-105 exhibits impairment in glucose catabolite repression of alcohol oxidase (AOX) synthesis resulting in the induction of AOX in a glucose containing growth medium [22]. Stable transformants bearing *UOX *under control of the AOX promoter with increased UOX enzyme activity were screened by a plate patches assay in solid glucose-containing medium. Finally, six positive recombinant strains (20, 22, 24, 28, 32, and 36) were selected (Figure 1B).

**Figure 2:**
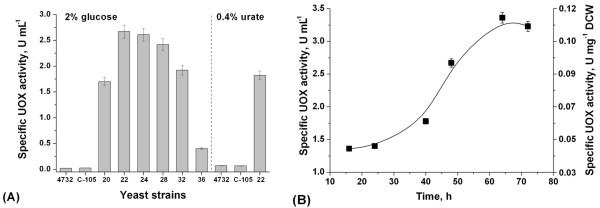
A: Specific UOX activity (U mL^-1^) in cell-free extracts of the initial strain and the recombinant strains grown in mineral media supplemented with 2% (wt/vol) glucose or 0.4% (wt/vol) urate; B: Time profile of UOX activity (U mL^-1 ^or U mg^-1 ^DCW) in cell-free extracts of the recombinant strain 22 showing the dynamics of yeast growth in mineral medium supplemented with 2% (wt/vol) glucose.

The UOX activity in cell-free extracts of the selected strains was determined spectrophotometrically. As shown in Figure 2A, the UOX activity of the selected strains varied between 0.4 and 2.67 U mL^-1^, while the initial strain of *H. polymorpha *C-105 showed no significant enzyme activity under the same growth conditions. The C-105 cells were cultivated in urate containing medium for induction of uricase. The UOX activity of C-105 cultivated under inducing conditions was 0.067 U mL^-1^. Thus, the most active strain 22 showed a forty-fold higher UOX activity as compared to the parental strain.

**Figure 3:**
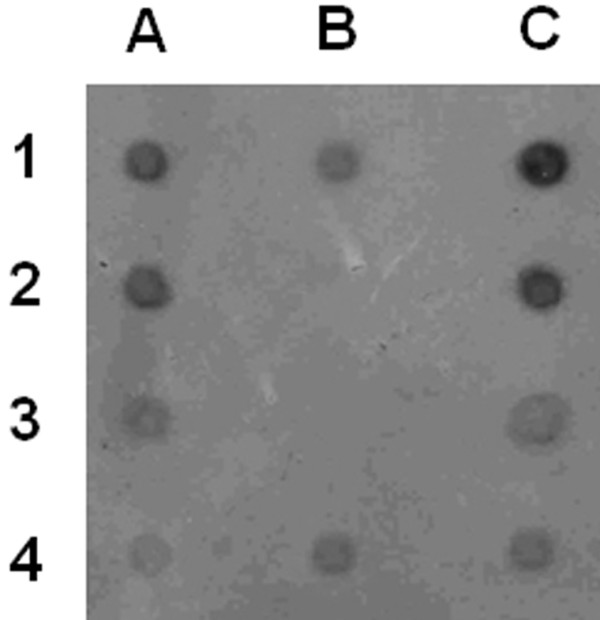
**Southern dot-blot of genomic DNAs of strains overproducing UOX probed with UOX ORF with terminator region**. Genomic DNAs of strains A--24 and C--22 with following DNA dilution: 1-- 1 μg without dilution; 2--twofold dilution; 3--5-fold dilution; 4--10-fold dilution. B/1 and 4 -- 1 μg of genomic DNA of parental strain C-105 without dilution (one copy control). The labeling of probe DNA and hybridization was performed using ECL direct nucleic acid labeling and detection system.

The vector used for construction of UOX producers, pGLG61_UOX, supports multiple tandem integration into the telomeric chromosome region [21]. The uricase gene copy number of strain 22 was established by quantitative Southern dot-blot via serial dilutions of the genomic DNA and comparing to untransformed strain's single-copy signal of the *H. polymorpha *uricase gene. The estimated vector copy number in transformants which are resistant to 1 mg mL^-1 ^G418 was approximately 10 (Figure 3).

The dependence of UOX activity of strain 22 on the growth phase is presented in Figure 2B. The maximal level of UOX activity in cell-free extracts occurred after 64 h of cell cultivation (3.36 U mL^-1 ^or 0.112 U mg^-1 ^DCW). This is in agreement with the dynamics of the development of the AOX activity in C-105 strain during cultivation in the same medium. As the yeast *H. polymorpha *belongs to the class of thermotolerant species with highest growth temperature around 50°C [23], the thermal stability of UOX was examined by incubating a cell-free extract for 10 min at 50°C. Uricase lost about 32% of its activity and reached an activity of 2.3 U mL^-1 ^at 50°C. Obtained data is in accordance with the thermal stability of uricase of *C. utilis*. Treatment at 50°C caused about 60% of activity maximum in the crude extract of the yeast [24].

The constructed overproducing strains exhibit significantly lower UOX activity as compared with commercially available recombinant UOX from *Escherichia coli *or *Saccharomyces cerevisiae *[25,26]. However, their genetic background makes the constructed strains a unique ideal biorecognition element in microbial urate-selective amperometric biosensors. The block in catalase activity leads to the generation of H_2_O_2 _as a result of urate oxidation that can be easily detected amperometrically. A defect in the gene responsible for catalase synthesis forced the cells to develop a mechanism of H_2_O_2 _depletion through its extrusion from the cell [22,27]. H_2_O_2 _released from the cell facilitating its detection. The impairment in glucose repression of the constructed strains allows for the overproduction of UOX in a low-priced glucose-containing growth medium that does not required expensive inducers, i.e. uric acid [28].

### Evaluation and optimization of the urate biosensor

UOX catalyzes *in vivo *oxidation of uric acid in the presence of oxygen as an oxidizing agent producing allantoin and CO_2 _as oxidation products of uric acid and hydrogen peroxide as a reduction product of O_2_. The amperometric detection of uric acid can be performed by electrochemical oxidation of the produced H_2_O_2_. However, there is one serious problem which has to be solved in such sensors, namely that uric acid itself is oxidized at Pt, Au and carbon electrodes at the potentials necessary to oxidize H_2_O_2 _[29-31]. Amperometric detection of H_2_O_2 _using horseradish peroxidase (HRP) for its biocatalytic reduction to water has been proposed [30,31].

**Figure 4:**
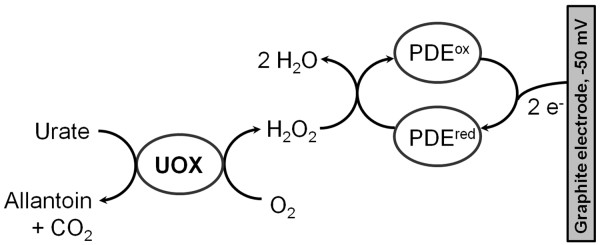
**Reaction scheme and electron-transfer pathway of the proposed urate biosensor**. PDE - hydrogen peroxide decomposing enzymes (HRP, CAT or MP-11). PDE^ox ^and PDE^red ^- oxidized and reduced forms of hydrogen peroxide decomposing enzymes, UOX - urate oxidase.

A bienzyme system comprising UOX and one of a hydrogen peroxide decomposing enzymes (HRP, catalase (CAT) or microperoxidase (MP-11)) was evaluated for the construction of urate-selective biosensors. The reaction scheme and electron-transfer pathway is presented in Figure 4.

**Figure 5:**
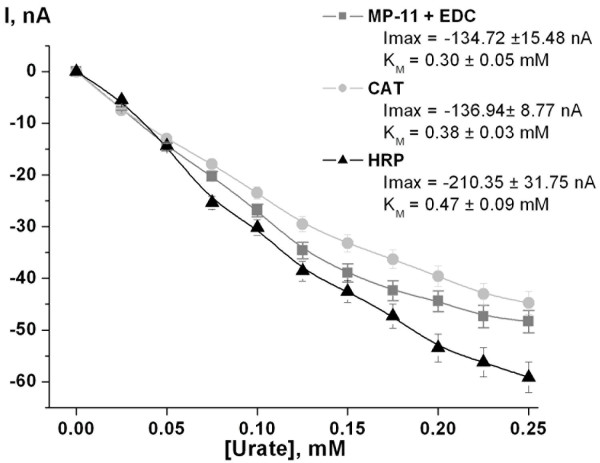
**Screening of the hydrogen peroxide decomposing enzymes (MP-11, CAT or HRP)**. 3.05 mm graphite rod electrodes, -50 mV vs. Ag/AgCl, 1 μL of each enzymes and 1 μL of cell suspension. PDE and suspension of recombinant cells were entrapped behind the dialysis membrane (cut off 10 kDa). Testing solution: 30 mM Tris-HCl (pH 8.9); working volume: 20 mL; stock solution of uric acid: 10 mM in 30 mM Tris-HCl (pH 8.9).

Biosensor based on living cells of the uricase-overproducing recombinant strain of *H. polymorpha *coupled with HRP exhibited the highest kinetic parameters I^max ^= 210 nA, K_M _= 0.47 mM for urate (Figure 5). Kinetic parameters of sensors based on CAT or MP-11 were quite similar (I^max ^= 137 or 135 nA, K_M _= 0.38 or 0.30 mM, respectively; Figure 5), however, due to the fact that the HRP-based sensors showed the highest K_M_-value all further investigations were done with HRP-based sensors. This feature provides a wider range of linearity thus improving the accuracy of the analysis by reducing of the necessary sample dilutions. Moreover the response time for the sensor based on HRP (1-1.2 min) was shorter than that for CAT (1.2-1.4 min) or MP-11 (1.5-1.8 min).

**Figure 6:**
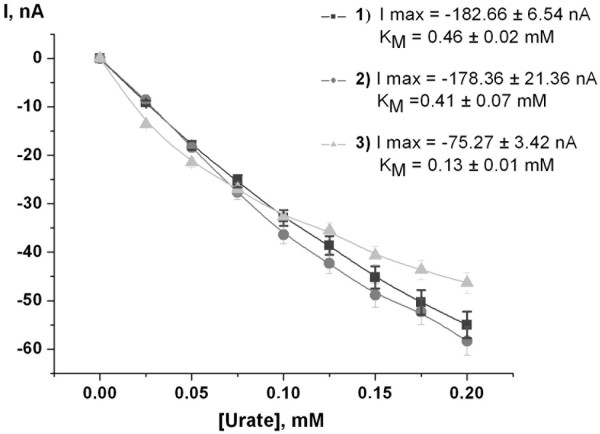
**Evaluation of the optimal enzyme ratio (3.05 mm graphite electrodes, -50 mV vs. Ag/AgCl)**. 1 μL HRP/1 μL of cell suspension - (■), 1.5 μL HRP/1 μL of cell suspension - (●), 2 μL HRP/1 μL of cell suspension - (▲). HRP and cell suspension were entrapped behind the dialysis membrane (cut off 10 kDa). Testing solution: 30 mM Tris-HCl (pH 8.9); working volume: 20 mL; stock solution of uric acid: 10 mM in 30 mM Tris-HCl (pH 8.9).

**Figure 7:**
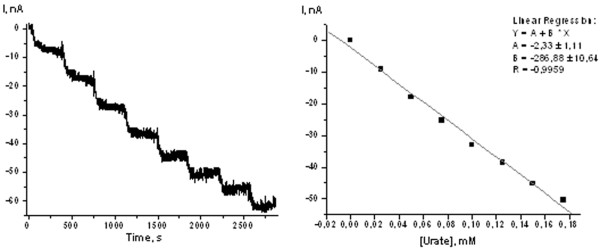
**Left: Chronoamperometric current response upon subsequent additions of urate aliquots obtained with a microbial biosensor entrapment behind a dialysis membrane; Right: urate calibration curve (-50 mV vs. Ag/AgCl)**. Testing solution: 30 mM Tris-HCl (pH 8.9); working volume: 20 mL; stock solution of uric acid: 10 mM in 30 mM Tris-HCl (pH 8.9).

The immobilization of the strain 22 cells on graphite disk electrodes was achieved by physical fixation behind dialysis membranes. For further optimization, the ratio of HRP to the cells and hence to UOX was varied. A combination of 1 μL of HRP solution and 1 μL of cell suspension showed the highest current response (Figure 6). The increase in the HRP/cell ratio let to a decrease of the current response apparently due to physical blocking of the electrode surface with HRP. The response time for the optimized sensor upon addition of 0.1 mM urate was about 5.5 nA min^-1^. A typical current response of a sensor with optimized architecture is shown in Figure 7. The detection limit of the sensor was determined to be 8 μM and the apparent K_M _value obtained from several independent calibrations was 0.46 mM for urate. The optimal pH range for the urate biosensor was 8.5 to 8.9, the optimal temperature range was between 36 to 42°C (data not shown).

**Figure 8:**
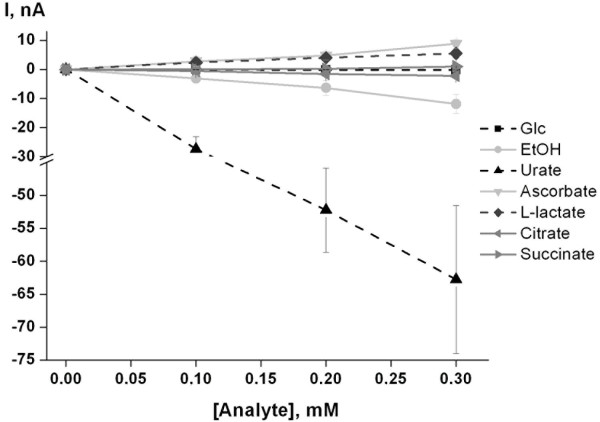
**Selectivity of the optimized biosensor**. Currents and relative responses of different concentrations of D-glucose, ethanol, urate, ascorbate, L-lactate, citrate and succinate are shown (-50 mV vs. Ag/AgCl). HRP and cell suspension were entrapped behind the dialysis membrane (cut off 10 kDa). Testing solution: 30 mM Tris-HCl (pH 8.9); working volume: 20 mL; stock solution of D-glucose, ethanol, urate, ascorbate, L-lactate, citrate and succinate: 10 mM in 30 mM Tris-HCl (pH 8.9). Sensor calibrations by possible urate interfering compounds were performed on the same electrodes. Urate calibration was performed after all measurements.

For application of the optimized prototype of the urate sensor in real samples, its selectivity with respect to potential interferences such as ascorbate, citrate, L-lactate, succinate, D-glucose, and ethanol is of great importance. Hence, the current response of the urate biosensor was evaluated with respect to these substances (Figure 8). The biosensor exhibits about 10% of cross-sensitivity to ethanol and ascorbate relative to the urate output level. However, no relative cross sensitivity to the other investigated compounds was observed. As the interfering compounds are present in significantly lower concentrations *e.g. *in urine, the impact of these compounds on the determination of urate in most real samples is assumed to be negligible.

The main characteristics of the developed urate selective microbial biosensors are comparable with previously reported results, such as an UOX-microperoxidase bi-enzyme system based on Au-electrode [13] or a redox mediator (FcCOOH) containing UOX-HRP system based on graphite electrode [12]. The detection limit is higher (8 μM compared to 2 μM or 0.6 μM of urate), while the linear detection range is wider (180 μM versus 75 μM or 120 μM of urate). The K_M_-values [13] coincide with our results. At the same time the estimated costs of the biosensor based on the urate selective microbial cells appears to be significantly reduced.

## Conclusions

A strain of *H. polymorpha *overproducing UOX was constructed. Genomic integration of expression cassettes of the uricase gene under the control of the strong *H. polymorpha *AOX promoter resulted in a 40-fold increase in the UOX activity compared to the parental strain. A method for preliminary screening of the recombinant strains was developed. The conditions for strain cultivation were optimized to ensure the maximal amount of the target enzyme.

The strain identified as the one possessing highest UOX activity was used for the construction of amperometric urate biosensors. A urate-selective biorecognition layer was designed comprising living cells of the recombinant *H. polymorpha *strain coupled to HRP. The terminal amperometric detection was achieved by direct electron transfer between HRP and the graphite electrode at -50 mV vs. Ag/AgCl. The sensor design was optimized and a ratio of HRP/cells of 1:1 yielded the highest current response. The detection limit of the optimized sensor design was 8 μM and the apparent K_M_-value 0.46 mM. Future research will be directed to further increasing the UOX activity of *H. polymorpha *and optimization of the electron-transfer cascade of the described bi-enzyme system.

## Materials and methods

### Materials

Urate, L-lactic acid, horseradish peroxidase (HRP, EC 1.11.1.7), microperoxidase-11 (MP-11), 1-ethyl-3-(3-dimethylaminopropyl)carbodiimide (EDC), catalase (CAT, EC 1.11.1.6) and uricase (UOX, EC 1.7.3.3) were obtained from Sigma. Succinic acid was from Fluka. Citric acid monohydrate, ascorbic acid, D(+)-glucose monohydrate were from J.T. Baker. Ethanol (abs.) was from Riedel-de Haën and EDTA was from Serva; (NH_4_)_2_SO_4_, Na_2_HPO_4_, KH_2_PO_4_, MgSO_4_, CaCl_2 _were obtained from Merck. Dialysis membranes (cut off 10 kDa) were from Biomol. All chemicals were of analytical grade and all solutions were prepared using HPLC-grade water. Solutions of glucose, ethanol, urate, L-lactic acid, ascorbic acid, succinic acid and citric acid were prepared in 30 mM Tris-HCl buffer, pH 8.9.

### Strains, media, cultivation and preparation of microbial cells

Cultivation of *H. polymorpha *CBS 4732 *(leu2-2) *[32], C-105 (*gcr1 catX*) [22] and the recombinant producer of uricase was performed in flasks on a shaker (200 rpm) at 37°C in a medium containing (g L^-1^): (NH_4_)_2_SO_4 _- 3.5; KH_2_PO_4 _- 1.0; MgSO_4 _× 7H_2_O - 0.5; CaCl_2 _- 0.1; yeast extract - 6. Glucose (20 g L^-1^) was used as carbon source. For induction of uricase, the cells from the mid-exponential growth phase were washed once in the mineral medium and transferred to shake-flask cultures supplemented with 4 g L^-1 ^urate as carbon source. Cultivation of the recombinant strain 22 was performed for 64 h. To prevent degradation of target enzyme, after washing, the cells were suspended in 30 mM Tris-HCl buffer (pH 8.9) containing 1 mM phenylmethylsulfonyl fluoride (PMSF) and 1 mM ethylenediaminetetraacetic acid (EDTA) followed by lyophilization. Before experiments, the lyophilized yeast cells were re-suspended to 30 mg mL^-1 ^dry cell weight (DCW) in 30 mM Tris-HCl buffer, pH 8.9, containing 1 mM EDTA. Cell-free extracts were obtained by vortexing with glass beads (425-600 μm; Sigma Cat No. G-8772) followed by centrifugation as described previously [33].

The yeast C-105 (*gcr1 catX*) was grown on YPS (10 g L^-1 ^yeast extract, 20 g L^-1 ^peptone, and 20 g L^-1 ^sucrose) at 37°C and used as a recipient strain for transformation experiments. For the selection of yeast transformants on YPS, 0.5-1.5 mg mL^-1 ^of the antibiotic geneticin (G418) was added. The *E. coli *strain DH5α (Φ80d*lacZ*ΔM15, *recA*1, *endA*1, *gyrA*96, *thi*-1, *hsdR*17(r_K_^-^, m_K_^+^), *supE*44, *relA*1, *deoR*, Δ(*lacZYA-argF*)U169) was used as a host for propagation of plasmids. The strain DH5α was grown at 37°C in LB medium as described previously [33]. Transformed *E. coli *cells were maintained on a medium containing 100 mg L^-1 ^ampicillin.

### Plasmid construction and molecular techniques

The *H. polymorpha *uricase gene with a terminator region (orf 201 hp_contig08, in the *H. polymorpha *genome database, Rhein Biotech) and the alcohol oxidase gene promoter were amplified from the genomic DNA of *H. polymorpha *strain CBS 4732 using the corresponding pairs of primers: Ko189 5'-CAA TCT AAA GTA CAA AAA CAA AGG TAC CAT GGC TGT CCT GCA ATC GTC-3' (*Kpn*I)/Ko190 5'-CCG GGA TCC TAC TCT TTG ATT GCC TCC-3' (*Bam*HI) and Ko183 CGC GGA TCC TCG TTT AGA ACG TCC TG-3' (*Bam*HI)/Ko188 5'-GAC GAT TGC AGG ACA GCC ATG GTA CCT TTG TTT TTG TAC TTT AGA TTG-3' (*Kpn*I). The primers Ko183 and Ko190 were used to obtain a ~2.7 kb fragment containing the *H. polymorpha *uricase gene with a terminator region driven by the alcohol oxidase gene promoter by overlap PCR. This fragment was treated with restriction endonuclease *Bam*HI and cloned into the *Bam*HI-linearized and dephosphorylated plasmid pGLG61 [21], resulting in the recombinant constructs pGLG61_UOX (Figure 1A).

Standard cloning techniques were applied [33]. PCR-amplification of the fragments of interest was done with Platinum^® ^*Taq *DNA Polymerase High Fidelity (Invitrogen) according to the manufacturer specification. PCR was performed in a GeneAmp^® ^PCR System 9700 thermocycler (Applied Biosystems). Transformation of the yeast *H. polymorpha *by electroporation was carried out as described previously [34]. Preparation of the total DNA from yeast was carried out by using the DNeasy^® ^Tissue Kit (Qiagen). Plasmid DNA isolations from *E. coli *were performed by using NucleoSpin^® ^Plasmid (Macherey-Nagel).

For quantitative Southern dot-blot, preparations of serial dilutions of yeast genomic DNAs were denatured in 0.4 M NaOH, spotted 2 μL per dot onto dry nylon membrane (Hybond N+, Amersham Pharmacia Biotech). The labeling of probe DNA and hybridization was performed using non-radioactive ECL direct nucleic acid labeling and detection system (Amersham Pharmacia Biotech) according to the manufacturer's manual.

### Urate oxidase assay

The UOX activity in cell-free extracts was determined spectrophotometrically by following the decrease of the absorbance at 293 nm. Assay mixtures contained 0.1 mM urate in 0.1 M Tris-HC1 buffer (pH 8.9) [35]. The reaction was started by the addition of the cell extract. Enzyme assays were performed at 25°C. One unit was defined as the amount of enzyme necessary to transform 1 μmol of urate into allantoin in 1 min at 25°C and pH 8.9.

Strains with elevated UOX activity were screened by a plate patch assay. UOX activity was visualized by the rate of H_2_O_2 _formation. H_2_O_2 _is formed in the reaction of UOX with urate and can be monitored by the peroxidative oxidation of *o*-dianisidine in the presence of horseradish peroxidase (HRP) leading to a purple colouring of the patches. The transformants were patched onto mineral medium agar plates supplemented with glucose. After 18 h of incubation at 37°C, the plates were overlaid with 9 mL of the UOX activity assay mixture containing 0.1 M Tris-HC1 buffer (pH 8.9), 0.7% (wt/vol) agar, digitonin (1 mg mL^-1^), *o*-dianisidine (0.5 mg mL^-1^), HRP Sigma RZ 1.1 (0.13 mg mL^-1^), and urate (0.1 mM). The plates were incubated at 37°C for up to 1 h. Patches that stained faster were selected for further experiments. All assay experiments were repeated at least twice.

### Preparation and evaluation of the cell-based biosensors

Amperometric cell-based biosensors were evaluated using constant-potential amperometry in a three-electrode configuration with an Ag/AgCl/KCl (3 M) reference electrode and a Pt-wire counter electrode. Amperometric measurements were carried out using a bipotentiostat (EP 30, Biometra, Göttingen, Germany) connected to a personal computer via a RS232 port for data acquisition. Graphite rods (type RW001, 3.05 mm diameter, Ringsdorff Werke, Bonn, Germany) were used as working electrodes. They were sealed in glass tubes by means of epoxy glue exposing a graphite disk. The electrodes were polished on emery paper and ultrasonicated in water.

HRP solution (300 U mL^-1^) was dropped on the electrode surface and dried at 4°C for 15 min. Alternative CAT solution (400 U mL^-1^) or MP-11 (0.05 mM) containing EDC (2.5 mM) were used. Amount of hydrogen peroxide decomposing enzymes was 1 μL, unless otherwise stated. Then, 1 μL of the freshly prepared cells were dropped on top of the hydrogen peroxide decomposing enzymatic layer (HRP, CAT or MP-11) and dried at 4°C for 15 min. Physical fixation was performed with a piece of dialysis membrane and an O-ring. Before use, the electrodes were rinsed with 30 mM Tris-HCl buffer, pH 8.9. Defined amount of analyte was injected to measuring cell after sensor stabilization (current baseline). The time of the sensor-system stabilization was 9-10 min. The additional of the next urate concentration was performed for the identical time periods (for approximately 5 min). The electrodes were washed with distilled water between measurements. Measurements were repeated at least three times to ensure reproducibility. Between experiments, the cell-modified electrodes were stored in buffer at 4°C.

## Authors' contributions

KVD carried out the molecular genetic studies, drafted and edited the manuscript. OVS and KVD constructed and characterized the amperometric biosensor. OVD performed the biochemical studies. OVS, WS and AAS co-drafted the manuscript. WS and AAS supervised some of the work and participated in the design of the experiments. All authors read and approved the final version of the manuscript.
